# Safety assessment of the pyridoindole derivative SMe1EC2: developmental neurotoxicity study in rats

**DOI:** 10.2478/v10102-011-0009-7

**Published:** 2011-03

**Authors:** Eduard Ujházy, Mojmír Mach, Jana Navarová, Ingrid Brucknerová, Michal Dubovický

**Affiliations:** 1 Institute of Experimental Pharmacology & Toxicology, Slovak Academy of Sciences, SK-84104 Bratislava, Slovak Republic; 2 1st Department of Pediatrics, Medical School, Comenius University, SK-83340 Bratislava, Slovak Republic

**Keywords:** pyridoindole drugs, brain development, behavior, developmental neurotoxicity, rat

## Abstract

The present study deals with effect of prenatal and neonatal administration of the synthetic pyridoindole derivative SMe1EC2 (2-ethoxycarbonyl-8-methoxy-2,3,4,4a,5,9b-hexahydro-1*H*-pyrido-[4,3b] indolinium chloride) on postnatal and neurobehavioral development of the rat offspring. The substance tested was administered to pregnant rats orally in the doses 5, 50 and 250 mg/kg from day 15 of gestation to day 10 *post partum* (PP). From the day 4 PP, the postnatal development and neurobehavioral characteritics of offspring were evaluated. The following variables were observed: body weight, pinna detachment, incisor eruption, ear opening, eye opening, testes descent and vaginal opening, righting reflex, negative geotaxia, startle reflex, dynamic air righting and exploratory behavior in a new environment. No maternal death, abortion or dead fetuses occurred either in the control or SMe1EC2 groups. Dynamic righting reflex was delayed one day in the groups of animals treated *via* their mothers with 5 and 50 mg/kg SMe1EC2. The delay in the development of this reflex was only transient. On day 20 PP, all pups tested had a positive score of the reflex. Administration of SMe1EC2 did not reveal any significant changes in other variables of somatic growth and maturation, reflex and neuromotor development and exploratory behavior, either of young or adult animals of both genders, assessed by analysis of variance.

## Introduction

Chemical substances as well as biological and physical factors can adversely affect the developing organism. Abnormal embryo-fetal development becomes manifest at various levels, ranging from death of the embryo/fetus, through serious congenital malformations to minor structural anomalies (Hood, 2006). Low doses of chemicals without any signs of maternal and embryo-fetal toxicity may interfere with developmental processes in the brain during prenatal as well as early postnatal periods. Functional maldevelopment of the brain, in turn, may result in neurological, behavioral, emotional and mental disorders, such as attention deficit-hyperactivity disorder, autism or schizophrenia (Cannon & Clarke, 2005; Kinney *et al*., 2008).

Hypoxia/ischemia associated with oxidative stress during prenatal and perinatal periods affects central nervous, cardiovascular, respiratory, gastrointestinal, hemopoetic and uropoetic systems, as well as the adrenal glands and the skin (Brucknerová, 2000). The most serious consequence is however injury of the developing brain manifested as hypoxic-ischemic encephalopathy, brain edema, bleeding to brain tissue, *etc.* (Dennery, 2007; Verklan, 2009).

Hypoxia/ischemia brain injury in newborns can be therapeutically affected by means of substances with antioxidant and antiradical properties, such as vitamin C, α-tocopherol, trolox, lazaroids, selenoids, allopurinol, calcium antagonists and calcium channel blockers (Penn *et al*., 1997; van Bel *et al*., 1998; Brucknerová, 2000; Cohen-Kerem & Koren, 2003). Pyridoindole derivatives of the parental drug stobadine designed and synthesized at the Institute of Experimental Pharmacology and Toxicology, Slovak Academy of Sciences, Bratislava, Slovakia, represent prospective drugs in the therapy of hypoxia/ischemia in the developing organism. The most thoroughly studied pyridoindole derivative is 2-ethoxycarbonyl-8-methoxy-2,3,4,4a,5,9b-hexahydro-1*H*-pyrido-[4,3b] indolinium chloride, m.w. 312.79 Da, chemical purity >99% (SMe1EC2). The results of preclinical experimental studies showed significant antioxidant and neuroprotective effects of SMe1EC2. This pyrodindole derivative has a very low toxicity, its LD_50_ is higher than 2400 mg/kg (Štolc *et al*., 2008). The results of a teratological study did not show any signs of maternal and embryofetal toxicity (Ujházy *et al*., 2008). As the parental drug stobadine showed protective effects on hypoxia induced injuries in the developing organism (Ujházy *et al*., 2004, 2006), it is possible to use the substance SMe1EC2 in the protection of embryos/fetuses and/or neonates against hypoxia/ischemia. However, every drug considered to be used during sensitive developmental stages must undergo preclinical safety assessment procedures. It is inevitable to have comprehensive knowledge on any possible adverse effects of the drug tested on the developing organism.

The aim of this experimental study was to investigate the effect of prenatal and neonatal administration of SMe1EC2 on postnatal development of the rat offspring focused on neurobehavioral development.

## Material and methods

### Developmental neurotoxicity study

In the present study we followed OECD Guideline No. 426 (OECD, 2007). It is based on evaluation of the effect of the substance tested during sensitive developmental stages of the brain on the postnatal and neurobehavioral development up to the adulthood.

### Animals

Virgin female Wistar/DV rats (n=69) aged 3–4 months, weight 200–220 g, and male rats (n=10), aged 3–4 months, weight 240–260 g obtained from the breeding station Dobrá Voda, Slovakia, were used. The animals were housed under standard experimental conditions (temperature 21±2 °C, relative humidity 55±10%, 12/12hr light-dark cycle, food and water provided *ad libitum*). After 7 days of acclimatization, the females were mated with males in the ratio 1 male: 3 females (presence of spermatozoa in vaginal smear indicated day 0 of gestation). Pregnant females were allowed to spontaneously deliver their pups (day 0 *post partum,* PP). On day 4 PP, the number of pups in each litter was reduced to 8 pups, 4 males and 4 females. The pups were weaned on day 21PP. The experiment was performed in compliance with the Principles of Laboratory Animal Care issued by the Ethical Committee of the Institute of Experimental Pharmacology and Toxicology, Slovak Academy of Sciences. The experimental design was approved by the State Veterinary and Food Administration of the Slovak Republic.

### Treatment

The substance tested was administered to pregnant rats orally in the doses of 5, 50 and 250 mg/kg from day 15 of gestation to day 10 PP. The substance was dissolved in saline at aconstant dosage volume 0.5 ml/100 g body weight. Control animals received vehiculum at the same time schedule.

### Maternal observation

During administration of the substance tested to pregnat rats, the following signs of possible intoxication were observed: lacrimation, salivation, piloerection, exophthalmos, urination, defecation, mydriasis, ptosis, tremor, convulsions, abnormal movements and behavior, cachexia, dehydratation, hypotony or hypertony and fur changes. Further variables observed were body weight gains of pregnant females, duration of pregnancy, number of live and dead pups.

### Postnatal development of offspring

#### Somatic growth and maturation

From day 4 PP we evaluated postnatal and neurobehavioral development of offspring. We observed the following variables of somatic growth and maturation. Body weight (days 4, 7, 14 and 21 PP), pinna detachment (day 4 PP), incisor eruption (day 10 PP), ear opening (day 12 PP), eye opening (day 15 PP), testes descent (day 27 PP) and vaginal opening (day 35 PP).

#### Neuromotor and reflex development

Within the neuromotor and reflex development assessment, we observed **righting reflex** (on day 5 PP, the pup′s ability to turn over from supine position), **negative geotaxia** (8 PP, the pup′s ability to turn 180° on a 25° inclined placed head down), **startle reflex** (15 PP, the presence or absence of sensorimotor reaction – jerks to auditory stimulus expressed as apercentage of pups with negative reaction), **dynamic air righting** (15–20 PP, the pup′s ability to turn over in the air from supine position and fall down onto 4 limbs).

#### Activity and emotional reactivity

Activity of the animals was recorded as exploratory behavior in anew environment (open field test). Young animals were tested by means of optoelectronic device ActiTrack (Panlab, Spain) on days 23–26 PP. The rat was placed in the center of the experimental glass box sized 43×43 cm. Adult animals were tested in the experimental arena with awith dimensions 40×60 cm on days 90–93 PP and analysed by videotracking software ANY-Maze (Stoelting Co., USA). Young as well as adult rats were tested on four consecutive days in 5 min sessions between 8.00–12.00 A.M. After each individual test, the number of fecal boli left were recorded and the open field arena was cleaned with amild detergent.

### Statistics

Data were analyzed by means of analysis of variance. Data from repeated testings were analyzed by “repeated measures ANOVA”. Data from single tests were analyzed by one-way ANOVA. The values are presented as means±S.E.M. The confidence limit of *p*<0.05 was considered statistically significant.

## Results

### Maternal toxicity

**Figure 1:**
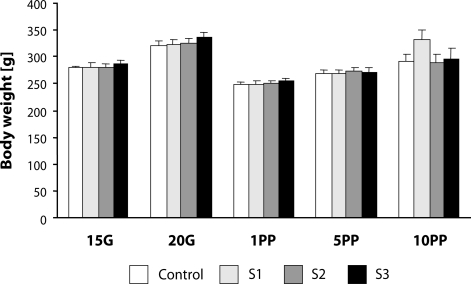
Effect of developmental administration of SMe1EC2 on body weight gain in pregnant females. Doses: S1 – 5 mg/kg, S2 – 50 mg/kg, S3 – 250 mg/kg, G – gestation, PP – *post partum*.

No maternal death, abortion or dead fetuses occurred either in the control or SMe1EC2 treated groups. The maternal body weight gains were not affected by the treatment with the substance tested (data not shown). There was only a significant effect of time on body weight gain, *i.e.* the body weight of mothers significantly changed during pregnancy and after delivery [F(4,144)=60.704; *p*<0.0001] (Figure 1).

### Effects on offspring

**Figure 2:**
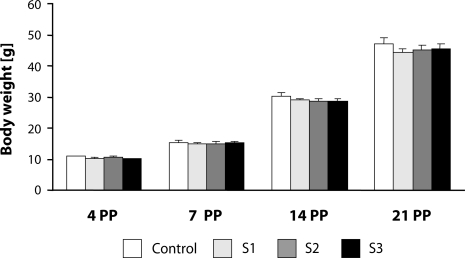
Effect of developmental administration of SMe1EC2 on body weight of male offspring. Doses: S1 – 5 mg/kg, S2 – 50 mg/kg, S3 – 250 mg/kg, PP – *post partum*.

Body weight of offspring of either gender was not affected by SMe1EC2. In all experimental groups of both genders there was a significant gradual increase in body weight from day 4 to day 21 PP of experiment [F(3,219)=50.273; *p<*0.0001] (Figure 2 males, females data not shown).

**Figure 3:**
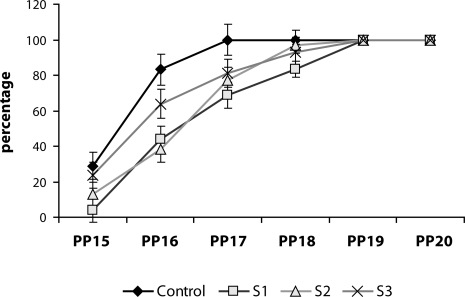
Effect of developmental administration of SMe1EC2 on dynamic righting reflex in male offspring. Doses: S1 – 5 mg/kg, S2 – 50 mg/kg, S3 – 250 mg/kg, PP – *post partum*. Effect of treatment: F(3,25)=7.043, *p<*0.01, on day PP 16, S1 and S2 groups compared to control – Fisher LSD post-hoc, *p<*0.001.

Individual variables of somatic growth and maturation were not affected by the treatment with SMe1EC2. Similarly there was no significant effect of the substance tested on the variables of the neuromotor and reflex development, except the dynamic air righting. During the test, there was a significant increase in the percentage of pups with positive score in both genders [F_males_(5,125)=144.92; *p*<0.0001, F_females_(5,125)=134.73; *p*<0.0001]. In males, there was a statistically significant effect of SMe1EC2 treatment on this variable [F(3,25)=7.043; *p*<0.01]. Fisher LSD post-hoc test revealed a significant decrease (*p*<0.001) in the percentage in the dose groups 5 and 50 mg/kg SMe1EC2 on day 16 PP compared to controls (Figure 3).

Analysis of variance did not reveal any significant changes in exploratory behavior of young or adult animals of either gender due to administration of SMe1EC2 during development (data not shown).

## Discussion

Prenatal and perinatal periods are highly vulnerable to the action of various chemical substances and physical factors. It is inevitable for the healthy organism to maintain homeostasis and in the case of its perturbation to recover as soon as possible the former physiological status. Hypoxia/ischemia followed by production of reactive oxygen species and oxidative stress is considered the most important factor for disturbation of homeostasis in the body (Hood, 2006; Dennery, 2007). Injuries induced by hypoxia/ischemia associated with oxidative stress in pregnancy and during parturition can be ameliorated by the use of natural products as well as synthetic drugs with antioxidant and antiradical properties. Experimental studies showed that treatment with antioxidants, such as vitamin C and E or butylated hydroxytoluene, can diminish embryofetal dysmorphogenesis due to gestational diabetes induced by hyperinsulinemia and hypoxia (Eriksson and Simán, 1996; Ponce *et al*., 2005; Cederberg and Eriksson, 2005). Moreover, vitamin C in pregnancy may play arole in providing an antioxidant defense against reactive oxygen species affecting birth weight (Park *et al*., 2004). Melatonin seems to be involved in correcting the pathophysiology of complications during pregnancy, including those due to abortion, pre-eclampsia and fetal brain damage (Tamura *et al*., 2008). Our previous experimental studies showed that pretreatment of pregnant rats with stobadine prevented to acertain extent reproductive and fetal developmental alterations due to chronic intrauterine hypoxia induced by phenytoin (Ujházy *et al*., 2004). In the study of neonatal anoxia, stobadine reduced anoxia-induced hyperactivity in male offspring (Ujházy *et al*., 2006). Gáspárová *et al*. (2010) found that maternal treatment with SMe1EC2 improved resistance of the offspring hippocampus against transient ischemia *in vitro*. This was documented by improved recovery of electrically evoked neuronal response in reoxygenation.

However, it is important to note that relatively high doses of the antioxidants are needed to normalize the development of offspring in experimental pathophysiological conditions. Yet treatment whith such high doses may also have adverse effects on intact animals (Cedeberg and Eriksson, 2005).

Concerning the synthetic pyridoindole derivative SMe1EC2 and its parental drug stobadine, series of preclinical relative safety studies were conducted in the past. In a teratological study on rats and mice, no signs of maternal and wembryofetal toxicity were found (Ujházy *et al*., 1992; 1994). Similarly, stobadine in a long-term toxicity and genotoxicity study (Gajdošíková *et al*., 1995) as well as in reproductive and behavioral studies (Balonová *et al*., 1991; Dubovický *et al*., 1999) did not exhibit any adverse effects.

In our present preclinical study, we investigated the relative safety of SMe1EC2 after its administration to pregnant and lactating rats. The results showed that the substance tested did not cause any negative effects on somatic and neurobehavioral development of offspring until adulthood. The only significant effect of the treatment was found in the dynamic righting reflex. The appearence of this reflex was delayed by one day in the groups of animals treated *via* their mothers with 5 and 50 mg/kg SMe1EC2. However, the delay of this reflex was only transient. On day 20 PP all pups tested had a positive score of the reflex. We did not consider this subtle transient alteration to be due to the adverse effect of the substance tested. The delay may be explained by different times of delivery. Pups delivered late at night were assigned the next day in the morning as their 0 PP day. This may have caused the one day shift in the development of this reflex.

The developmental neurotoxicity study based on the OECD Guideline No. 426 (2007) recommends to administer the chemical substance during pregnancy and lactation and to evaluate postnatal development up to adulthood. Investigation of somatic growth and maturation, reflex and neuromotor development, sensory function, activity and emotional reactivity, memory and learning processes should be performed within the study. However, recent studies showed that other behavioral characteristics, such as anxiety, depression, abnormal social behavior or altered stress responsiveness can occur due to developmental exposure to various factors during the postnatal period (Ferguson *et al*., 2009; Braquenier *et al*., 2010.). These changes may manifest at late adulthood or even in senescence or in reaction to stressful stimuli (Makatsori *et al*., 2005). In our opinion, the battery of tests for chemical substances based on the OECD Guideline No. 426 (2007) does not involve all behavioral characteristics. Testing of social and reproductive behavior, anxiety and/or depression and reactivity to stressful stimuli should be included in this kind of studies. Thus also with the pyridoindole SMe1EC2 furher behavioral tests have to be conducted.

In conclusion, the results of this study showed a relative safety of SMe1EC2 on postnatal development of rat offspring from the point of view of somatic growth and maturation, reflex and neuromotor development, as well as exploratory behavior in a new environment.
